# Bromido{dicyclo­hexyl[2′-(dimethyl­amino)biphenyl-2-yl]phosphine-κ*P*}[2-(4,6-dimethyl­pyrimidin-2-yl)ferrocenyl-κ^2^ *C* ^1^,*N*]palladium(II) dichloro­methane solvate

**DOI:** 10.1107/S1600536809012884

**Published:** 2009-04-18

**Authors:** Xin-Qi Hao, Hong-Mei Li, Hui Jiang, Yong-Tao Ma, Mao-Ping Song

**Affiliations:** a Department of Chemistry, Henan Key Laboratory of Chemical Biology and Organic Chemistry, Zhengzhou University, Zhengzhou 450052, People’s Republic of China; b Luoyang Normal University Library, Luoyang 471022, People’s Republic of China

## Abstract

In the title compound, [FePdBr(C_5_H_5_)(C_11_H_10_N_2_)(C_26_H_36_NP)]·CH_2_Cl_2_, the Pd atom displays a distorted square-planar coordination environment. The five-membered metallacycle adopts an envelope conformation with the coordinated cyclo­penta­dienyl C atom 0.4222 (4) Å out of plane. The dihedral angle between the pyrimidinyl ring and substituted cyclo­penta­dienyl ring is 21.47 (2)°. In the crystal structure, the dimeric unit is generated through the C—H⋯π contact *via* a crystallographic inversion centre, while the C—H⋯Cl contacts in the dimeric centre link the dichlormethane mol­ecules with the Pd complex mol­ecules.

## Related literature

For historical background of cyclo­palladated compounds, see: Cope & Sickman (1965 ▶). For the properties of cyclo­palladated compounds, see: Dupont *et al.* (2005 ▶); Gong *et al.* (2007 ▶); Xu *et al.* (2007 ▶). For related structures, see: Xu *et al.* (2008 ▶, 2009 ▶).
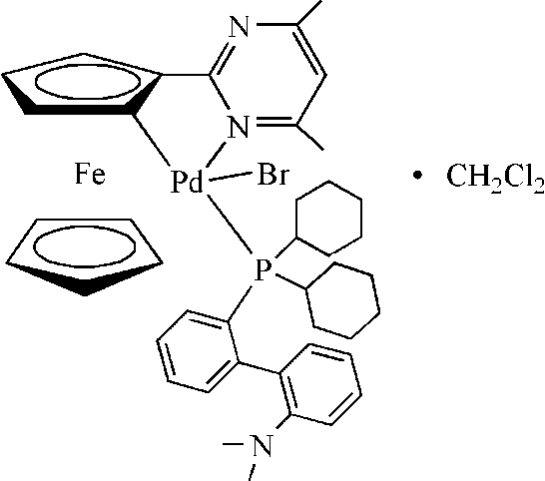


## Experimental

### 

#### Crystal data

[FePdBr(C_5_H_5_)(C_11_H_10_N_2_)(C_26_H_36_NP)]·CH_2_Cl_2_
*M* *_r_* = 955.91
Triclinic, 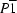

*a* = 10.2059 (9) Å
*b* = 10.3637 (9) Å
*c* = 21.900 (2) Å
α = 81.195 (1)°
β = 76.590 (1)°
γ = 72.647 (1)°
*V* = 2141.9 (3) Å^3^
*Z* = 2
Mo *K*α radiation
μ = 1.89 mm^−1^
*T* = 293 K
0.37 × 0.24 × 0.21 mm

#### Data collection

Bruker SMART APEX CCD area-detector diffractometer
Absorption correction: multi-scan (*SADABS*; Sheldrick, 1996 ▶) *T*_min_ = 0.540, *T*_max_ = 0.697
16458 measured reflections
7919 independent reflections
6716 reflections with *I* > 2σ(*I*)
*R*_int_ = 0.017

#### Refinement

*R*[*F* ^2^ > 2σ(*F* ^2^)] = 0.038
*wR*(*F* ^2^) = 0.099
*S* = 1.08
7919 reflections
473 parameters
H-atom parameters constrained
Δρ_max_ = 1.03 e Å^−3^
Δρ_min_ = −0.69 e Å^−3^

### 

Data collection: *APEX2* (Bruker, 2004 ▶); cell refinement: *SAINT* (Bruker, 2004 ▶); data reduction: *SAINT*; program(s) used to solve structure: *SHELXS97* (Sheldrick, 2008 ▶); program(s) used to refine structure: *SHELXL97* (Sheldrick, 2008 ▶); molecular graphics: *SHELXTL* (Sheldrick, 2008 ▶); software used to prepare material for publication: *SHELXTL*.

## Supplementary Material

Crystal structure: contains datablocks global, I. DOI: 10.1107/S1600536809012884/si2165sup1.cif

Structure factors: contains datablocks I. DOI: 10.1107/S1600536809012884/si2165Isup2.hkl

Additional supplementary materials:  crystallographic information; 3D view; checkCIF report

## Figures and Tables

**Table:** 

Pd1—C1	1.997 (3)
Pd1—N1	2.192 (3)
Pd1—P1	2.2654 (9)
Pd1—Br1	2.6585 (6)

**Table:** 

N1—Pd1—P1	173.00 (9)
C1—Pd1—Br1	160.40 (10)

**Table 2:** Hydrogen-bond geometry (Å, °)

*D*—H⋯*A*	*D*—H	H⋯*A*	*D*⋯*A*	*D*—H⋯*A*
C3—H3⋯Cl2^i^	0.98	2.78	3.671 (5)	151
C16—H16*C*⋯*Cg*1^ii^	0.96	2.68	3.544 (6)	151

Symmetry codes: (i) 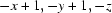; (ii) 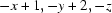. *Cg*1 is the centroid of the C6–C10 Cp ring.

**Fig. 1.**
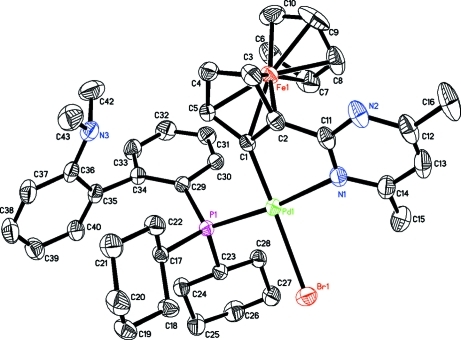
The molecular structure of the title compound with displacement ellipsoids at the 30% probability level. The dichloromethane molecule is omitted for clarity.

**Fig. 2.**
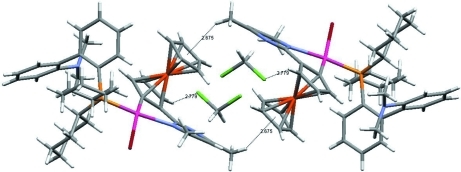
The dimeric unit of the title compound formed by the intermolecular C—H···Cl hydrogen bonds and C—H···π interactions. Cg1 is the centroid of the Cp ring C6—C10, symmetry code ii: 1 - x, 2 - y, -z.

## supplementary crystallographic information

### Comment

Cyclopalladated compounds containing a Pd—C bond intramolecularly stabilized by one donor atom were first reported in the middle 1960's (Cope & Sickman, 1965). Forty years later, the chemistry of these compound has developed into one of the most fruitful fields in organometallic chemistry (Dupont *et al.*, 2005). In the palladium-catalyzed coupling reactions, palladacycles have shown enormous superiority in many respects (Gong *et al.*, 2007; Xu *et al.*, 2007).

The Pd atom in the title complex is in a distorted square-planar environment bonded to the atoms shown in Fig. 1: the Br atom,the pyrimidinyl nitrogen atom and the carbon atom of the ferrocenyl moiety. The Pd—C1 (N1,P1,Br1) bond lengths and the P1—Pd1—N1 and C1—Pd1—Br1 bond angles (Table 1) are within comparable ranges of 162.08 (7) ° - 174.76 (7) ° and 162.27 (9) ° - 166.04 (8) ° (C—Pd—Cl), respectively (Xu *et al.*, 2009). The five-membered metallacycle (containing atoms C1/C2/C11/N1/Pd1) adopts an envelope conformation, with the C1 atom 0.4222 (4) Å out of plane. The dihedral angle between the pyrimidinyl ring and substituted cyclopentadienyl ring is 21.47 (2)°. In the crystal structure, the dimeric unit (Fig.2) is generated through the C—H···π contacts *via* a crystallographic inversion centre, and the C—H···Cl contact (Table 2) connects a symmetry-related dichlormethane molecule with the Pd complex (Table 2).This hydrogen bonding motif is different from those of related cyclopalladated ferrocene derivatives (Xu *et al.*, 2008; Xu *et al.*, 2009), in which C—H···Cl hydrogen bonds construct the one-dimensional chain structures.

### Experimental

The title compound was prepared as described in literature (Xu *et al.*, 2009), using Li_2_PdBr_4_ instead of Li_2_PdCl_4_ and recrystallized from dichloromethane-petroleum ether solution at room temperature to give the desired product as red crystals suitable for single-crystal X-ray diffraction.

### Refinement

H atoms attached to C atoms of the title compound were placed in geometrically idealized positions and treated as riding with C—H distances constrained to 0.93–0.96 Å, and with *U*_iso_(H)=1.2*U*_eq_(C) (1.5*U*_eq_ for methyl H).

### Crystal data

**Table:**

[FePdBr(C_5_H_5_)(C_11_H_10_N_2_)(C_26_H_36_NP)]·CH_2_Cl_2_	*Z* = 2
*M**_r_* = 955.91	*F*(000) = 976
Triclinic, *P*1	*D*_x_ = 1.482 Mg m^−^^3^
*a* = 10.2059 (9) Å	Mo *K*α radiation, λ = 0.71073 Å
*b* = 10.3637 (9) Å	Cell parameters from 7107 reflections
*c* = 21.900 (2) Å	θ = 2.5–27.1°
α = 81.195 (1)°	µ = 1.89 mm^−^^1^
β = 76.590 (1)°	*T* = 293 K
γ = 72.647 (1)°	Block, red
*V* = 2141.9 (3) Å^3^	0.37 × 0.24 × 0.21 mm

### Data collection

**Table:**

Bruker SMART APEX CCD area-detector diffractometer	7919 independent reflections
Radiation source: fine-focus sealed tube	6716 reflections with *I* > 2σ(*I*)
graphite	*R*_int_ = 0.017
φ and ω scans	θ_max_ = 25.5°, θ_min_ = 2.5°
Absorption correction: multi-scan (*SADABS*; Sheldrick, 1996)	*h* = −12→10
*T*_min_ = 0.540, *T*_max_ = 0.697	*k* = −12→12
16458 measured reflections	*l* = −26→26

### Refinement

**Table:**

Refinement on *F*^2^	Primary atom site location: structure-invariant direct methods
Least-squares matrix: full	Secondary atom site location: difference Fourier map
*R*[*F*^2^ > 2σ(*F*^2^)] = 0.038	Hydrogen site location: inferred from neighbouring sites
*wR*(*F*^2^) = 0.099	H-atom parameters constrained
*S* = 1.08	*w* = 1/[σ^2^(*F*_o_^2^) + (0.0421*P*)^2^ + 2.3428*P*] where *P* = (*F*_o_^2^ + 2*F*_c_^2^)/3
7919 reflections	(Δ/σ)_max_ = 0.001
473 parameters	Δρ_max_ = 1.03 e Å^−^^3^
0 restraints	Δρ_min_ = −0.69 e Å^−^^3^

### Special details

**Table:**

Geometry. All e.s.d.'s (except the e.s.d. in the dihedral angle between two l.s. planes)are estimated using the full covariance matrix. The cell e.s.d.'s are takeninto account individually in the estimation of e.s.d.'s in distances, anglesand torsion angles; correlations between e.s.d.'s in cell parameters are onlyused when they are defined by crystal symmetry. An approximate (isotropic)treatment of cell e.s.d.'s is used for estimating e.s.d.'s involving l.s. planes.
Refinement. Refinement of *F*^2^ against ALL reflections. The weighted *R*-factor *wR* and goodness of fit *S* are based on *F*^2^, conventional *R*-factors *R* are based on *F*, with *F* set to zero for negative *F*^2^. The threshold expression of *F*^2^ > σ(*F*^2^) is used only for calculating *R*-factors(gt) *etc*. and is not relevant to the choice of reflections for refinement. *R*-factors based on *F*^2^ are statistically about twice as large as those based on *F*, and *R*- factors based on ALL data will be even larger.

### Fractional atomic coordinates and isotropic or equivalent isotropic displacement parameters (Å^2^)

**Table:**

	*x*	*y*	*z*	*U*_iso_*/*U*_eq_	
Pd1	0.29216 (3)	0.66381 (3)	0.217306 (12)	0.03942 (9)	
Fe1	0.63058 (6)	0.70760 (5)	0.12158 (2)	0.04439 (14)	
P1	0.33910 (9)	0.56740 (8)	0.31270 (4)	0.03503 (19)	
Br1	0.02020 (5)	0.68171 (6)	0.25105 (2)	0.07101 (15)	
Cl2	0.1066 (2)	0.5000 (3)	0.07189 (12)	0.1586 (9)	
Cl3	0.2456 (4)	0.2376 (3)	0.12552 (12)	0.1980 (14)	
N1	0.2744 (3)	0.7531 (3)	0.12117 (14)	0.0477 (7)	
N2	0.3968 (4)	0.7000 (3)	0.01637 (15)	0.0592 (9)	
N3	0.7663 (3)	0.2896 (3)	0.29096 (16)	0.0565 (8)	
C1	0.4926 (4)	0.6039 (3)	0.17412 (16)	0.0395 (7)	
C2	0.5018 (4)	0.6044 (3)	0.10732 (16)	0.0443 (8)	
C3	0.6430 (4)	0.5430 (4)	0.07875 (18)	0.0537 (10)	
H3	0.6788	0.5297	0.0339	0.064*	
C4	0.7212 (4)	0.5024 (4)	0.1271 (2)	0.0544 (10)	
H4	0.8219	0.4579	0.1214	0.065*	
C5	0.6299 (4)	0.5404 (3)	0.18529 (18)	0.0449 (8)	
H5	0.6578	0.5268	0.2262	0.054*	
C6	0.6949 (6)	0.8426 (5)	0.1587 (2)	0.0754 (14)	
H6	0.7205	0.8302	0.2001	0.091*	
C7	0.5625 (6)	0.9016 (4)	0.1460 (3)	0.0746 (14)	
H7	0.4782	0.9384	0.1771	0.090*	
C8	0.5682 (7)	0.9016 (5)	0.0818 (3)	0.0812 (16)	
H8	0.4892	0.9383	0.0601	0.097*	
C9	0.7057 (8)	0.8393 (6)	0.0538 (3)	0.096 (2)	
H9	0.7411	0.8256	0.0090	0.115*	
C10	0.7859 (5)	0.8021 (5)	0.1023 (3)	0.0872 (17)	
H10	0.8866	0.7577	0.0970	0.105*	
C11	0.3860 (4)	0.6874 (4)	0.07887 (17)	0.0477 (9)	
C12	0.2921 (6)	0.7939 (5)	−0.0063 (2)	0.0674 (13)	
C13	0.1860 (6)	0.8765 (5)	0.0328 (2)	0.0724 (13)	
H13	0.1202	0.9473	0.0159	0.087*	
C14	0.1768 (5)	0.8542 (4)	0.0977 (2)	0.0618 (11)	
C15	0.0679 (5)	0.9476 (5)	0.1408 (3)	0.0829 (15)	
H15A	−0.0102	0.9103	0.1570	0.124*	
H15B	0.0371	1.0344	0.1180	0.124*	
H15C	0.1064	0.9585	0.1751	0.124*	
C16	0.3020 (7)	0.8073 (5)	−0.0767 (2)	0.0927 (19)	
H16A	0.2361	0.7675	−0.0862	0.139*	
H16B	0.3951	0.7613	−0.0967	0.139*	
H16C	0.2812	0.9016	−0.0921	0.139*	
C17	0.3460 (4)	0.3850 (3)	0.32533 (16)	0.0397 (8)	
H17	0.3978	0.3440	0.3592	0.048*	
C18	0.2011 (4)	0.3582 (4)	0.3453 (2)	0.0540 (10)	
H18A	0.1514	0.3996	0.3839	0.065*	
H18B	0.1467	0.3982	0.3128	0.065*	
C19	0.2181 (5)	0.2047 (4)	0.3555 (2)	0.0662 (12)	
H19A	0.2673	0.1663	0.3899	0.079*	
H19B	0.1264	0.1885	0.3674	0.079*	
C20	0.2977 (5)	0.1351 (4)	0.2974 (2)	0.0716 (13)	
H20A	0.2446	0.1673	0.2640	0.086*	
H20B	0.3099	0.0380	0.3062	0.086*	
C21	0.4392 (5)	0.1628 (4)	0.2760 (2)	0.0682 (12)	
H21A	0.4859	0.1223	0.2368	0.082*	
H21B	0.4962	0.1206	0.3073	0.082*	
C22	0.4266 (4)	0.3154 (4)	0.26618 (19)	0.0518 (9)	
H22A	0.5194	0.3291	0.2551	0.062*	
H22B	0.3789	0.3559	0.2315	0.062*	
C23	0.1984 (4)	0.6508 (3)	0.37623 (16)	0.0409 (8)	
H23	0.1134	0.6308	0.3716	0.049*	
C24	0.2157 (4)	0.5979 (4)	0.44399 (17)	0.0508 (9)	
H24A	0.2947	0.6205	0.4527	0.061*	
H24B	0.2339	0.4999	0.4491	0.061*	
C25	0.0821 (5)	0.6618 (5)	0.4903 (2)	0.0681 (13)	
H25A	0.0948	0.6303	0.5331	0.082*	
H25B	0.0047	0.6332	0.4835	0.082*	
C26	0.0467 (5)	0.8173 (5)	0.4817 (2)	0.0737 (13)	
H26A	−0.0408	0.8554	0.5098	0.088*	
H26B	0.1196	0.8466	0.4926	0.088*	
C27	0.0340 (5)	0.8691 (4)	0.4137 (2)	0.0623 (11)	
H27A	−0.0448	0.8474	0.4042	0.075*	
H27B	0.0166	0.9670	0.4085	0.075*	
C28	0.1661 (4)	0.8057 (4)	0.36824 (19)	0.0503 (9)	
H28A	0.2440	0.8324	0.3758	0.060*	
H28B	0.1547	0.8385	0.3254	0.060*	
C29	0.4941 (3)	0.5949 (3)	0.33234 (15)	0.0372 (7)	
C30	0.5028 (4)	0.7283 (3)	0.31298 (17)	0.0443 (8)	
H30	0.4448	0.7823	0.2864	0.053*	
C31	0.5932 (4)	0.7823 (4)	0.3316 (2)	0.0554 (10)	
H31	0.5953	0.8713	0.3178	0.066*	
C32	0.6802 (5)	0.7048 (5)	0.3703 (2)	0.0632 (11)	
H32	0.7396	0.7413	0.3845	0.076*	
C33	0.6785 (5)	0.5717 (4)	0.3881 (2)	0.0609 (11)	
H33	0.7388	0.5194	0.4140	0.073*	
C34	0.5909 (4)	0.5118 (4)	0.36905 (17)	0.0438 (8)	
C35	0.6087 (4)	0.3642 (4)	0.39042 (18)	0.0460 (8)	
C36	0.6959 (4)	0.2591 (4)	0.3527 (2)	0.0526 (9)	
C37	0.7073 (5)	0.1256 (4)	0.3780 (2)	0.0662 (12)	
H37	0.7620	0.0554	0.3536	0.079*	
C38	0.6395 (6)	0.0938 (5)	0.4387 (3)	0.0771 (14)	
H38	0.6486	0.0035	0.4542	0.092*	
C39	0.5598 (5)	0.1945 (5)	0.4756 (2)	0.0699 (12)	
H39	0.5159	0.1734	0.5165	0.084*	
C40	0.5445 (4)	0.3289 (4)	0.45172 (19)	0.0553 (10)	
H40	0.4900	0.3973	0.4772	0.066*	
C42	0.8764 (5)	0.3547 (6)	0.2856 (3)	0.0867 (16)	
H42A	0.8416	0.4319	0.3099	0.130*	
H42B	0.9064	0.3839	0.2422	0.130*	
H42C	0.9541	0.2915	0.3012	0.130*	
C43	0.8130 (6)	0.1821 (5)	0.2488 (3)	0.0862 (16)	
H43A	0.8887	0.1117	0.2625	0.129*	
H43B	0.8445	0.2183	0.2068	0.129*	
H43C	0.7368	0.1453	0.2495	0.129*	
C44	0.2410 (10)	0.4080 (8)	0.1087 (4)	0.138 (3)	
H44A	0.2313	0.4467	0.1477	0.166*	
H44B	0.3292	0.4154	0.0820	0.166*	

### Atomic displacement parameters (Å^2^)

**Table:**

	*U*^11^	*U*^22^	*U*^33^	*U*^12^	*U*^13^	*U*^23^
Pd1	0.03597 (15)	0.04066 (15)	0.04152 (15)	−0.01016 (11)	−0.01180 (11)	0.00234 (11)
Fe1	0.0491 (3)	0.0377 (3)	0.0443 (3)	−0.0144 (2)	−0.0046 (2)	0.0003 (2)
P1	0.0351 (4)	0.0315 (4)	0.0382 (4)	−0.0091 (3)	−0.0079 (3)	−0.0011 (3)
Br1	0.0486 (2)	0.0991 (4)	0.0643 (3)	−0.0267 (2)	−0.0149 (2)	0.0144 (2)
Cl2	0.0879 (13)	0.193 (2)	0.161 (2)	−0.0153 (13)	0.0182 (13)	−0.0262 (17)
Cl3	0.345 (5)	0.142 (2)	0.1273 (18)	−0.095 (2)	−0.063 (2)	0.0086 (15)
N1	0.0528 (19)	0.0448 (17)	0.0487 (18)	−0.0164 (15)	−0.0190 (15)	0.0061 (14)
N2	0.096 (3)	0.0469 (19)	0.0463 (19)	−0.0302 (19)	−0.0288 (18)	0.0041 (15)
N3	0.0462 (18)	0.0532 (19)	0.062 (2)	−0.0014 (15)	−0.0091 (16)	−0.0063 (16)
C1	0.0429 (19)	0.0334 (17)	0.0402 (18)	−0.0112 (15)	−0.0069 (15)	0.0024 (14)
C2	0.059 (2)	0.0326 (17)	0.0422 (19)	−0.0151 (16)	−0.0081 (17)	−0.0029 (15)
C3	0.070 (3)	0.039 (2)	0.045 (2)	−0.0119 (18)	0.0007 (19)	−0.0070 (16)
C4	0.047 (2)	0.041 (2)	0.065 (3)	−0.0057 (17)	0.0009 (19)	−0.0053 (18)
C5	0.043 (2)	0.0377 (19)	0.047 (2)	−0.0068 (15)	−0.0078 (16)	0.0053 (15)
C6	0.107 (4)	0.071 (3)	0.069 (3)	−0.053 (3)	−0.028 (3)	0.007 (2)
C7	0.094 (4)	0.040 (2)	0.089 (4)	−0.027 (2)	−0.003 (3)	−0.011 (2)
C8	0.117 (5)	0.046 (3)	0.093 (4)	−0.034 (3)	−0.050 (4)	0.025 (3)
C9	0.159 (6)	0.076 (4)	0.058 (3)	−0.075 (4)	0.020 (4)	−0.004 (3)
C10	0.057 (3)	0.066 (3)	0.136 (5)	−0.030 (2)	−0.002 (3)	−0.001 (3)
C11	0.069 (3)	0.0349 (18)	0.047 (2)	−0.0231 (18)	−0.0209 (19)	0.0027 (16)
C12	0.112 (4)	0.050 (2)	0.060 (3)	−0.042 (3)	−0.045 (3)	0.016 (2)
C13	0.092 (4)	0.058 (3)	0.078 (3)	−0.023 (3)	−0.052 (3)	0.019 (2)
C14	0.063 (3)	0.054 (2)	0.074 (3)	−0.018 (2)	−0.034 (2)	0.011 (2)
C15	0.067 (3)	0.067 (3)	0.100 (4)	0.005 (2)	−0.025 (3)	0.003 (3)
C16	0.177 (6)	0.064 (3)	0.063 (3)	−0.052 (4)	−0.066 (4)	0.018 (2)
C17	0.0454 (19)	0.0312 (17)	0.0453 (19)	−0.0144 (15)	−0.0129 (15)	0.0022 (14)
C18	0.056 (2)	0.049 (2)	0.062 (2)	−0.0258 (19)	−0.0076 (19)	−0.0030 (18)
C19	0.073 (3)	0.053 (2)	0.080 (3)	−0.036 (2)	−0.014 (2)	0.006 (2)
C20	0.090 (3)	0.042 (2)	0.093 (4)	−0.030 (2)	−0.022 (3)	−0.008 (2)
C21	0.076 (3)	0.038 (2)	0.089 (3)	−0.015 (2)	−0.008 (3)	−0.013 (2)
C22	0.057 (2)	0.040 (2)	0.057 (2)	−0.0152 (17)	−0.0027 (18)	−0.0091 (17)
C23	0.0379 (18)	0.0372 (18)	0.0446 (19)	−0.0077 (14)	−0.0058 (15)	−0.0026 (15)
C24	0.055 (2)	0.047 (2)	0.043 (2)	−0.0045 (18)	−0.0075 (17)	−0.0050 (16)
C25	0.071 (3)	0.067 (3)	0.046 (2)	0.003 (2)	−0.002 (2)	−0.003 (2)
C26	0.068 (3)	0.069 (3)	0.069 (3)	0.001 (2)	0.004 (2)	−0.025 (2)
C27	0.062 (3)	0.037 (2)	0.074 (3)	0.0025 (18)	−0.002 (2)	−0.0109 (19)
C28	0.053 (2)	0.0358 (19)	0.057 (2)	−0.0084 (17)	−0.0060 (18)	−0.0027 (16)
C29	0.0369 (17)	0.0375 (17)	0.0375 (17)	−0.0094 (14)	−0.0087 (14)	−0.0040 (14)
C30	0.045 (2)	0.0369 (18)	0.052 (2)	−0.0125 (16)	−0.0096 (16)	−0.0033 (16)
C31	0.061 (2)	0.046 (2)	0.065 (3)	−0.0234 (19)	−0.012 (2)	−0.0048 (19)
C32	0.063 (3)	0.063 (3)	0.079 (3)	−0.030 (2)	−0.024 (2)	−0.013 (2)
C33	0.059 (3)	0.063 (3)	0.069 (3)	−0.018 (2)	−0.032 (2)	0.001 (2)
C34	0.045 (2)	0.0404 (19)	0.045 (2)	−0.0093 (16)	−0.0131 (16)	−0.0013 (15)
C35	0.045 (2)	0.042 (2)	0.052 (2)	−0.0058 (16)	−0.0220 (17)	0.0005 (16)
C36	0.045 (2)	0.047 (2)	0.062 (2)	−0.0005 (17)	−0.0219 (18)	0.0005 (18)
C37	0.068 (3)	0.045 (2)	0.076 (3)	0.001 (2)	−0.021 (2)	0.000 (2)
C38	0.088 (4)	0.047 (3)	0.088 (4)	−0.009 (2)	−0.026 (3)	0.012 (2)
C39	0.081 (3)	0.065 (3)	0.062 (3)	−0.022 (3)	−0.019 (2)	0.011 (2)
C40	0.062 (3)	0.054 (2)	0.050 (2)	−0.013 (2)	−0.0198 (19)	0.0008 (18)
C42	0.051 (3)	0.109 (4)	0.098 (4)	−0.023 (3)	−0.007 (3)	−0.011 (3)
C43	0.085 (4)	0.076 (3)	0.082 (4)	0.000 (3)	−0.001 (3)	−0.025 (3)
C44	0.198 (9)	0.139 (7)	0.125 (6)	−0.101 (6)	−0.080 (6)	0.033 (5)

### Geometric parameters (Å, °)

**Table:**

Pd1—C1	1.997 (3)	C18—H18A	0.9700
Pd1—N1	2.192 (3)	C18—H18B	0.9700
Pd1—P1	2.2654 (9)	C19—C20	1.501 (6)
Pd1—Br1	2.6585 (6)	C19—H19A	0.9700
Fe1—C2	2.025 (4)	C19—H19B	0.9700
Fe1—C3	2.028 (4)	C20—C21	1.510 (6)
Fe1—C9	2.032 (5)	C20—H20A	0.9700
Fe1—C7	2.034 (4)	C20—H20B	0.9700
Fe1—C10	2.035 (5)	C21—C22	1.533 (5)
Fe1—C8	2.041 (4)	C21—H21A	0.9700
Fe1—C4	2.047 (4)	C21—H21B	0.9700
Fe1—C6	2.047 (5)	C22—H22A	0.9700
Fe1—C5	2.053 (3)	C22—H22B	0.9700
Fe1—C1	2.058 (3)	C23—C28	1.530 (5)
P1—C29	1.841 (3)	C23—C24	1.532 (5)
P1—C17	1.851 (3)	C23—H23	0.9800
P1—C23	1.857 (3)	C24—C25	1.533 (5)
Cl2—C44	1.715 (8)	C24—H24A	0.9700
Cl3—C44	1.737 (7)	C24—H24B	0.9700
N1—C14	1.343 (5)	C25—C26	1.535 (6)
N1—C11	1.364 (5)	C25—H25A	0.9700
N2—C11	1.337 (5)	C25—H25B	0.9700
N2—C12	1.346 (6)	C26—C27	1.525 (6)
N3—C36	1.414 (5)	C26—H26A	0.9700
N3—C42	1.449 (6)	C26—H26B	0.9700
N3—C43	1.454 (6)	C27—C28	1.513 (5)
C1—C5	1.420 (5)	C27—H27A	0.9700
C1—C2	1.443 (5)	C27—H27B	0.9700
C2—C3	1.425 (5)	C28—H28A	0.9700
C2—C11	1.448 (5)	C28—H28B	0.9700
C3—C4	1.411 (6)	C29—C30	1.405 (5)
C3—H3	0.9800	C29—C34	1.419 (5)
C4—C5	1.425 (5)	C30—C31	1.372 (5)
C4—H4	0.9800	C30—H30	0.9300
C5—H5	0.9800	C31—C32	1.368 (6)
C6—C7	1.381 (7)	C31—H31	0.9300
C6—C10	1.398 (8)	C32—C33	1.379 (6)
C6—H6	0.9800	C32—H32	0.9300
C7—C8	1.394 (7)	C33—C34	1.393 (5)
C7—H7	0.9800	C33—H33	0.9300
C8—C9	1.393 (8)	C34—C35	1.499 (5)
C8—H8	0.9800	C35—C40	1.396 (5)
C9—C10	1.427 (8)	C35—C36	1.420 (5)
C9—H9	0.9800	C36—C37	1.391 (6)
C10—H10	0.9800	C37—C38	1.389 (7)
C12—C13	1.371 (7)	C37—H37	0.9300
C12—C16	1.510 (6)	C38—C39	1.360 (7)
C13—C14	1.391 (6)	C38—H38	0.9300
C13—H13	0.9300	C39—C40	1.386 (6)
C14—C15	1.488 (7)	C39—H39	0.9300
C15—H15A	0.9600	C40—H40	0.9300
C15—H15B	0.9600	C42—H42A	0.9600
C15—H15C	0.9600	C42—H42B	0.9600
C16—H16A	0.9600	C42—H42C	0.9600
C16—H16B	0.9600	C43—H43A	0.9600
C16—H16C	0.9600	C43—H43B	0.9600
C17—C22	1.521 (5)	C43—H43C	0.9600
C17—C18	1.537 (5)	C44—H44A	0.9700
C17—H17	0.9800	C44—H44B	0.9700
C18—C19	1.534 (5)		
			
C1—Pd1—N1	80.12 (13)	C14—C15—H15B	109.5
C1—Pd1—P1	93.07 (10)	H15A—C15—H15B	109.5
N1—Pd1—P1	173.00 (9)	C14—C15—H15C	109.5
C1—Pd1—Br1	160.40 (10)	H15A—C15—H15C	109.5
N1—Pd1—Br1	92.84 (8)	H15B—C15—H15C	109.5
P1—Pd1—Br1	94.13 (3)	C12—C16—H16A	109.5
C2—Fe1—C3	41.18 (15)	C12—C16—H16B	109.5
C2—Fe1—C9	122.5 (2)	H16A—C16—H16B	109.5
C3—Fe1—C9	106.90 (19)	C12—C16—H16C	109.5
C2—Fe1—C7	123.4 (2)	H16A—C16—H16C	109.5
C3—Fe1—C7	159.1 (2)	H16B—C16—H16C	109.5
C9—Fe1—C7	67.4 (2)	C22—C17—C18	109.9 (3)
C2—Fe1—C10	159.3 (2)	C22—C17—P1	109.9 (2)
C3—Fe1—C10	122.8 (2)	C18—C17—P1	113.7 (2)
C9—Fe1—C10	41.1 (2)	C22—C17—H17	107.7
C7—Fe1—C10	67.2 (2)	C18—C17—H17	107.7
C2—Fe1—C8	107.86 (19)	P1—C17—H17	107.7
C3—Fe1—C8	122.8 (2)	C19—C18—C17	109.7 (3)
C9—Fe1—C8	40.0 (2)	C19—C18—H18A	109.7
C7—Fe1—C8	40.0 (2)	C17—C18—H18A	109.7
C10—Fe1—C8	67.7 (2)	C19—C18—H18B	109.7
C2—Fe1—C4	68.24 (16)	C17—C18—H18B	109.7
C3—Fe1—C4	40.50 (17)	H18A—C18—H18B	108.2
C9—Fe1—C4	123.1 (2)	C20—C19—C18	111.8 (4)
C7—Fe1—C4	159.5 (2)	C20—C19—H19A	109.3
C10—Fe1—C4	108.1 (2)	C18—C19—H19A	109.3
C8—Fe1—C4	158.8 (2)	C20—C19—H19B	109.3
C2—Fe1—C6	158.8 (2)	C18—C19—H19B	109.3
C3—Fe1—C6	159.1 (2)	H19A—C19—H19B	107.9
C9—Fe1—C6	67.9 (2)	C19—C20—C21	110.7 (4)
C7—Fe1—C6	39.6 (2)	C19—C20—H20A	109.5
C10—Fe1—C6	40.1 (2)	C21—C20—H20A	109.5
C8—Fe1—C6	67.1 (2)	C19—C20—H20B	109.5
C4—Fe1—C6	124.0 (2)	C21—C20—H20B	109.5
C2—Fe1—C5	68.29 (15)	H20A—C20—H20B	108.1
C3—Fe1—C5	68.69 (16)	C20—C21—C22	111.7 (4)
C9—Fe1—C5	159.4 (3)	C20—C21—H21A	109.3
C7—Fe1—C5	123.73 (19)	C22—C21—H21A	109.3
C10—Fe1—C5	123.2 (2)	C20—C21—H21B	109.3
C8—Fe1—C5	159.2 (2)	C22—C21—H21B	109.3
C4—Fe1—C5	40.68 (15)	H21A—C21—H21B	107.9
C6—Fe1—C5	108.64 (18)	C17—C22—C21	111.2 (3)
C2—Fe1—C1	41.39 (14)	C17—C22—H22A	109.4
C3—Fe1—C1	69.73 (15)	C21—C22—H22A	109.4
C9—Fe1—C1	158.8 (3)	C17—C22—H22B	109.4
C7—Fe1—C1	107.86 (17)	C21—C22—H22B	109.4
C10—Fe1—C1	158.2 (2)	H22A—C22—H22B	108.0
C8—Fe1—C1	123.1 (2)	C28—C23—C24	110.0 (3)
C4—Fe1—C1	68.68 (14)	C28—C23—P1	113.1 (2)
C6—Fe1—C1	122.59 (18)	C24—C23—P1	116.3 (2)
C5—Fe1—C1	40.43 (14)	C28—C23—H23	105.5
C29—P1—C17	110.59 (16)	C24—C23—H23	105.5
C29—P1—C23	101.08 (16)	P1—C23—H23	105.5
C17—P1—C23	106.19 (16)	C23—C24—C25	109.8 (3)
C29—P1—Pd1	115.27 (11)	C23—C24—H24A	109.7
C17—P1—Pd1	112.84 (12)	C25—C24—H24A	109.7
C23—P1—Pd1	109.86 (11)	C23—C24—H24B	109.7
C14—N1—C11	117.0 (3)	C25—C24—H24B	109.7
C14—N1—Pd1	132.7 (3)	H24A—C24—H24B	108.2
C11—N1—Pd1	110.3 (2)	C24—C25—C26	111.1 (4)
C11—N2—C12	116.1 (4)	C24—C25—H25A	109.4
C36—N3—C42	116.4 (4)	C26—C25—H25A	109.4
C36—N3—C43	116.8 (4)	C24—C25—H25B	109.4
C42—N3—C43	109.8 (4)	C26—C25—H25B	109.4
C5—C1—C2	106.1 (3)	H25A—C25—H25B	108.0
C5—C1—Pd1	142.9 (3)	C27—C26—C25	110.4 (4)
C2—C1—Pd1	109.9 (3)	C27—C26—H26A	109.6
C5—C1—Fe1	69.6 (2)	C25—C26—H26A	109.6
C2—C1—Fe1	68.06 (19)	C27—C26—H26B	109.6
Pd1—C1—Fe1	132.86 (17)	C25—C26—H26B	109.6
C3—C2—C1	109.1 (3)	H26A—C26—H26B	108.1
C3—C2—C11	130.0 (3)	C28—C27—C26	111.1 (3)
C1—C2—C11	119.3 (3)	C28—C27—H27A	109.4
C3—C2—Fe1	69.5 (2)	C26—C27—H27A	109.4
C1—C2—Fe1	70.6 (2)	C28—C27—H27B	109.4
C11—C2—Fe1	114.3 (2)	C26—C27—H27B	109.4
C4—C3—C2	107.3 (3)	H27A—C27—H27B	108.0
C4—C3—Fe1	70.5 (2)	C27—C28—C23	110.7 (3)
C2—C3—Fe1	69.3 (2)	C27—C28—H28A	109.5
C4—C3—H3	126.3	C23—C28—H28A	109.5
C2—C3—H3	126.3	C27—C28—H28B	109.5
Fe1—C3—H3	126.3	C23—C28—H28B	109.5
C3—C4—C5	108.6 (3)	H28A—C28—H28B	108.1
C3—C4—Fe1	69.0 (2)	C30—C29—C34	117.3 (3)
C5—C4—Fe1	69.9 (2)	C30—C29—P1	111.3 (3)
C3—C4—H4	125.7	C34—C29—P1	131.0 (3)
C5—C4—H4	125.7	C31—C30—C29	122.9 (4)
Fe1—C4—H4	125.7	C31—C30—H30	118.6
C1—C5—C4	109.0 (3)	C29—C30—H30	118.6
C1—C5—Fe1	69.99 (19)	C32—C31—C30	119.7 (4)
C4—C5—Fe1	69.5 (2)	C32—C31—H31	120.1
C1—C5—H5	125.5	C30—C31—H31	120.1
C4—C5—H5	125.5	C31—C32—C33	118.9 (4)
Fe1—C5—H5	125.5	C31—C32—H32	120.6
C7—C6—C10	108.1 (5)	C33—C32—H32	120.6
C7—C6—Fe1	69.7 (3)	C32—C33—C34	123.2 (4)
C10—C6—Fe1	69.5 (3)	C32—C33—H33	118.4
C7—C6—H6	125.9	C34—C33—H33	118.4
C10—C6—H6	125.9	C33—C34—C29	117.8 (3)
Fe1—C6—H6	125.9	C33—C34—C35	115.7 (3)
C6—C7—C8	109.0 (5)	C29—C34—C35	126.5 (3)
C6—C7—Fe1	70.8 (3)	C40—C35—C36	118.7 (4)
C8—C7—Fe1	70.3 (3)	C40—C35—C34	118.3 (3)
C6—C7—H7	125.5	C36—C35—C34	122.8 (3)
C8—C7—H7	125.5	C37—C36—N3	121.2 (4)
Fe1—C7—H7	125.5	C37—C36—C35	117.9 (4)
C9—C8—C7	108.1 (5)	N3—C36—C35	120.9 (3)
C9—C8—Fe1	69.7 (3)	C38—C37—C36	122.0 (4)
C7—C8—Fe1	69.7 (3)	C38—C37—H37	119.0
C9—C8—H8	125.9	C36—C37—H37	119.0
C7—C8—H8	125.9	C39—C38—C37	120.1 (4)
Fe1—C8—H8	125.9	C39—C38—H38	119.9
C8—C9—C10	107.2 (5)	C37—C38—H38	119.9
C8—C9—Fe1	70.3 (3)	C38—C39—C40	119.6 (4)
C10—C9—Fe1	69.6 (3)	C38—C39—H39	120.2
C8—C9—H9	126.4	C40—C39—H39	120.2
C10—C9—H9	126.4	C39—C40—C35	121.7 (4)
Fe1—C9—H9	126.4	C39—C40—H40	119.1
C6—C10—C9	107.5 (5)	C35—C40—H40	119.1
C6—C10—Fe1	70.4 (3)	N3—C42—H42A	109.5
C9—C10—Fe1	69.3 (3)	N3—C42—H42B	109.5
C6—C10—H10	126.3	H42A—C42—H42B	109.5
C9—C10—H10	126.3	N3—C42—H42C	109.5
Fe1—C10—H10	126.3	H42A—C42—H42C	109.5
N2—C11—N1	125.8 (4)	H42B—C42—H42C	109.5
N2—C11—C2	120.5 (4)	N3—C43—H43A	109.5
N1—C11—C2	113.6 (3)	N3—C43—H43B	109.5
N2—C12—C13	121.1 (4)	H43A—C43—H43B	109.5
N2—C12—C16	116.5 (5)	N3—C43—H43C	109.5
C13—C12—C16	122.3 (5)	H43A—C43—H43C	109.5
C12—C13—C14	120.0 (4)	H43B—C43—H43C	109.5
C12—C13—H13	120.0	Cl2—C44—Cl3	112.9 (4)
C14—C13—H13	120.0	Cl2—C44—H44A	109.0
N1—C14—C13	119.2 (4)	Cl3—C44—H44A	109.0
N1—C14—C15	119.8 (4)	Cl2—C44—H44B	109.0
C13—C14—C15	120.8 (4)	Cl3—C44—H44B	109.0
C14—C15—H15A	109.5	H44A—C44—H44B	107.8
			
C1—Pd1—P1—C29	43.93 (15)	C10—C6—C7—Fe1	59.0 (3)
N1—Pd1—P1—C29	31.0 (7)	C2—Fe1—C7—C6	162.9 (3)
Br1—Pd1—P1—C29	−154.31 (12)	C3—Fe1—C7—C6	−160.3 (4)
C1—Pd1—P1—C17	−84.43 (15)	C9—Fe1—C7—C6	−82.1 (4)
N1—Pd1—P1—C17	−97.3 (7)	C10—Fe1—C7—C6	−37.4 (3)
Br1—Pd1—P1—C17	77.32 (12)	C8—Fe1—C7—C6	−119.5 (5)
C1—Pd1—P1—C23	157.29 (15)	C4—Fe1—C7—C6	43.1 (7)
N1—Pd1—P1—C23	144.4 (7)	C5—Fe1—C7—C6	78.2 (3)
Br1—Pd1—P1—C23	−40.96 (12)	C1—Fe1—C7—C6	119.9 (3)
C1—Pd1—N1—C14	−159.6 (4)	C2—Fe1—C7—C8	−77.6 (4)
P1—Pd1—N1—C14	−146.5 (6)	C3—Fe1—C7—C8	−40.8 (7)
Br1—Pd1—N1—C14	38.9 (4)	C9—Fe1—C7—C8	37.3 (4)
C1—Pd1—N1—C11	21.8 (2)	C10—Fe1—C7—C8	82.1 (4)
P1—Pd1—N1—C11	34.9 (8)	C4—Fe1—C7—C8	162.6 (5)
Br1—Pd1—N1—C11	−139.8 (2)	C6—Fe1—C7—C8	119.5 (5)
N1—Pd1—C1—C5	172.1 (4)	C5—Fe1—C7—C8	−162.3 (3)
P1—Pd1—C1—C5	−6.3 (4)	C1—Fe1—C7—C8	−120.6 (3)
Br1—Pd1—C1—C5	−117.7 (4)	C6—C7—C8—C9	1.1 (5)
N1—Pd1—C1—C2	−22.0 (2)	Fe1—C7—C8—C9	−59.3 (3)
P1—Pd1—C1—C2	159.5 (2)	C6—C7—C8—Fe1	60.4 (3)
Br1—Pd1—C1—C2	48.1 (4)	C2—Fe1—C8—C9	−119.6 (4)
N1—Pd1—C1—Fe1	56.1 (2)	C3—Fe1—C8—C9	−76.7 (4)
P1—Pd1—C1—Fe1	−122.3 (2)	C7—Fe1—C8—C9	119.4 (5)
Br1—Pd1—C1—Fe1	126.2 (2)	C10—Fe1—C8—C9	38.9 (3)
C2—Fe1—C1—C5	118.0 (3)	C4—Fe1—C8—C9	−43.8 (7)
C3—Fe1—C1—C5	80.6 (2)	C6—Fe1—C8—C9	82.4 (4)
C9—Fe1—C1—C5	165.1 (5)	C5—Fe1—C8—C9	164.7 (5)
C7—Fe1—C1—C5	−121.4 (3)	C1—Fe1—C8—C9	−162.6 (3)
C10—Fe1—C1—C5	−48.4 (6)	C2—Fe1—C8—C7	121.0 (3)
C8—Fe1—C1—C5	−162.8 (3)	C3—Fe1—C8—C7	163.9 (3)
C4—Fe1—C1—C5	37.1 (2)	C9—Fe1—C8—C7	−119.4 (5)
C6—Fe1—C1—C5	−80.5 (3)	C10—Fe1—C8—C7	−80.5 (4)
C3—Fe1—C1—C2	−37.4 (2)	C4—Fe1—C8—C7	−163.2 (5)
C9—Fe1—C1—C2	47.1 (6)	C6—Fe1—C8—C7	−37.0 (3)
C7—Fe1—C1—C2	120.6 (3)	C5—Fe1—C8—C7	45.3 (7)
C10—Fe1—C1—C2	−166.4 (5)	C1—Fe1—C8—C7	78.0 (4)
C8—Fe1—C1—C2	79.3 (3)	C7—C8—C9—C10	−0.7 (5)
C4—Fe1—C1—C2	−80.8 (2)	Fe1—C8—C9—C10	−60.1 (3)
C6—Fe1—C1—C2	161.5 (3)	C7—C8—C9—Fe1	59.3 (3)
C5—Fe1—C1—C2	−118.0 (3)	C2—Fe1—C9—C8	78.9 (4)
C2—Fe1—C1—Pd1	−97.3 (3)	C3—Fe1—C9—C8	121.2 (3)
C3—Fe1—C1—Pd1	−134.7 (3)	C7—Fe1—C9—C8	−37.3 (3)
C9—Fe1—C1—Pd1	−50.2 (6)	C10—Fe1—C9—C8	−118.0 (5)
C7—Fe1—C1—Pd1	23.3 (3)	C4—Fe1—C9—C8	162.6 (3)
C10—Fe1—C1—Pd1	96.3 (5)	C6—Fe1—C9—C8	−80.3 (4)
C8—Fe1—C1—Pd1	−18.1 (3)	C5—Fe1—C9—C8	−164.5 (5)
C4—Fe1—C1—Pd1	−178.2 (3)	C1—Fe1—C9—C8	43.9 (7)
C6—Fe1—C1—Pd1	64.2 (3)	C2—Fe1—C9—C10	−163.1 (3)
C5—Fe1—C1—Pd1	144.7 (4)	C3—Fe1—C9—C10	−120.8 (3)
C5—C1—C2—C3	−0.4 (4)	C7—Fe1—C9—C10	80.6 (4)
Pd1—C1—C2—C3	−171.6 (2)	C8—Fe1—C9—C10	118.0 (5)
Fe1—C1—C2—C3	59.1 (3)	C4—Fe1—C9—C10	−79.4 (4)
C5—C1—C2—C11	−167.1 (3)	C6—Fe1—C9—C10	37.7 (3)
Pd1—C1—C2—C11	21.7 (4)	C5—Fe1—C9—C10	−46.5 (7)
Fe1—C1—C2—C11	−107.6 (3)	C1—Fe1—C9—C10	161.8 (4)
C5—C1—C2—Fe1	−59.5 (2)	C7—C6—C10—C9	0.6 (5)
Pd1—C1—C2—Fe1	129.34 (18)	Fe1—C6—C10—C9	59.7 (3)
C9—Fe1—C2—C3	78.2 (3)	C7—C6—C10—Fe1	−59.2 (3)
C7—Fe1—C2—C3	161.0 (3)	C8—C9—C10—C6	0.1 (5)
C10—Fe1—C2—C3	45.6 (6)	Fe1—C9—C10—C6	−60.4 (3)
C8—Fe1—C2—C3	119.7 (3)	C8—C9—C10—Fe1	60.5 (3)
C4—Fe1—C2—C3	−38.1 (2)	C2—Fe1—C10—C6	162.0 (5)
C6—Fe1—C2—C3	−167.7 (5)	C3—Fe1—C10—C6	−163.9 (3)
C5—Fe1—C2—C3	−82.0 (2)	C9—Fe1—C10—C6	118.3 (5)
C1—Fe1—C2—C3	−120.1 (3)	C7—Fe1—C10—C6	37.0 (3)
C3—Fe1—C2—C1	120.1 (3)	C8—Fe1—C10—C6	80.4 (4)
C9—Fe1—C2—C1	−161.7 (3)	C4—Fe1—C10—C6	−121.7 (3)
C7—Fe1—C2—C1	−78.9 (3)	C5—Fe1—C10—C6	−79.4 (4)
C10—Fe1—C2—C1	165.7 (5)	C1—Fe1—C10—C6	−44.0 (6)
C8—Fe1—C2—C1	−120.2 (3)	C2—Fe1—C10—C9	43.7 (7)
C4—Fe1—C2—C1	82.0 (2)	C3—Fe1—C10—C9	77.8 (4)
C6—Fe1—C2—C1	−47.6 (6)	C7—Fe1—C10—C9	−81.3 (4)
C5—Fe1—C2—C1	38.06 (19)	C8—Fe1—C10—C9	−37.9 (3)
C3—Fe1—C2—C11	−125.7 (4)	C4—Fe1—C10—C9	120.0 (3)
C9—Fe1—C2—C11	−47.5 (4)	C6—Fe1—C10—C9	−118.3 (5)
C7—Fe1—C2—C11	35.3 (4)	C5—Fe1—C10—C9	162.3 (3)
C10—Fe1—C2—C11	−80.1 (6)	C1—Fe1—C10—C9	−162.3 (4)
C8—Fe1—C2—C11	−6.0 (3)	C12—N2—C11—N1	5.3 (6)
C4—Fe1—C2—C11	−163.8 (3)	C12—N2—C11—C2	−170.8 (3)
C6—Fe1—C2—C11	66.6 (6)	C14—N1—C11—N2	−10.9 (5)
C5—Fe1—C2—C11	152.2 (3)	Pd1—N1—C11—N2	168.0 (3)
C1—Fe1—C2—C11	114.2 (3)	C14—N1—C11—C2	165.5 (3)
C1—C2—C3—C4	0.9 (4)	Pd1—N1—C11—C2	−15.6 (4)
C11—C2—C3—C4	165.6 (4)	C3—C2—C11—N2	10.2 (6)
Fe1—C2—C3—C4	60.5 (3)	C1—C2—C11—N2	173.6 (3)
C1—C2—C3—Fe1	−59.7 (2)	Fe1—C2—C11—N2	93.2 (4)
C11—C2—C3—Fe1	105.1 (4)	C3—C2—C11—N1	−166.4 (4)
C2—Fe1—C3—C4	−118.1 (3)	C1—C2—C11—N1	−3.0 (5)
C9—Fe1—C3—C4	121.5 (3)	Fe1—C2—C11—N1	−83.4 (3)
C7—Fe1—C3—C4	−167.6 (5)	C11—N2—C12—C13	3.9 (6)
C10—Fe1—C3—C4	79.4 (3)	C11—N2—C12—C16	−179.0 (4)
C8—Fe1—C3—C4	162.4 (3)	N2—C12—C13—C14	−7.1 (7)
C6—Fe1—C3—C4	49.4 (6)	C16—C12—C13—C14	175.9 (4)
C5—Fe1—C3—C4	−37.2 (2)	C11—N1—C14—C13	7.0 (6)
C1—Fe1—C3—C4	−80.5 (2)	Pd1—N1—C14—C13	−171.6 (3)
C9—Fe1—C3—C2	−120.3 (3)	C11—N1—C14—C15	−168.0 (4)
C7—Fe1—C3—C2	−49.5 (6)	Pd1—N1—C14—C15	13.5 (6)
C10—Fe1—C3—C2	−162.5 (3)	C12—C13—C14—N1	1.3 (7)
C8—Fe1—C3—C2	−79.5 (3)	C12—C13—C14—C15	176.2 (5)
C4—Fe1—C3—C2	118.1 (3)	C29—P1—C17—C22	−87.5 (3)
C6—Fe1—C3—C2	167.5 (5)	C23—P1—C17—C22	163.6 (3)
C5—Fe1—C3—C2	81.0 (2)	Pd1—P1—C17—C22	43.2 (3)
C1—Fe1—C3—C2	37.6 (2)	C29—P1—C17—C18	148.7 (3)
C2—C3—C4—C5	−1.0 (4)	C23—P1—C17—C18	39.9 (3)
Fe1—C3—C4—C5	58.8 (3)	Pd1—P1—C17—C18	−80.5 (3)
C2—C3—C4—Fe1	−59.8 (2)	C22—C17—C18—C19	57.1 (4)
C2—Fe1—C4—C3	38.7 (2)	P1—C17—C18—C19	−179.1 (3)
C9—Fe1—C4—C3	−76.8 (3)	C17—C18—C19—C20	−58.0 (5)
C7—Fe1—C4—C3	167.4 (5)	C18—C19—C20—C21	56.7 (5)
C10—Fe1—C4—C3	−119.6 (3)	C19—C20—C21—C22	−54.9 (5)
C8—Fe1—C4—C3	−44.7 (6)	C18—C17—C22—C21	−56.3 (4)
C6—Fe1—C4—C3	−160.9 (3)	P1—C17—C22—C21	177.7 (3)
C5—Fe1—C4—C3	120.3 (3)	C20—C21—C22—C17	55.5 (5)
C1—Fe1—C4—C3	83.4 (2)	C29—P1—C23—C28	71.5 (3)
C2—Fe1—C4—C5	−81.6 (2)	C17—P1—C23—C28	−173.0 (3)
C3—Fe1—C4—C5	−120.3 (3)	Pd1—P1—C23—C28	−50.7 (3)
C9—Fe1—C4—C5	163.0 (3)	C29—P1—C23—C24	−57.1 (3)
C7—Fe1—C4—C5	47.1 (6)	C17—P1—C23—C24	58.3 (3)
C10—Fe1—C4—C5	120.2 (3)	Pd1—P1—C23—C24	−179.3 (2)
C8—Fe1—C4—C5	−164.9 (5)	C28—C23—C24—C25	58.1 (4)
C6—Fe1—C4—C5	78.8 (3)	P1—C23—C24—C25	−171.8 (3)
C1—Fe1—C4—C5	−36.9 (2)	C23—C24—C25—C26	−57.2 (5)
C2—C1—C5—C4	−0.2 (4)	C24—C25—C26—C27	56.0 (5)
Pd1—C1—C5—C4	166.0 (3)	C25—C26—C27—C28	−56.1 (5)
Fe1—C1—C5—C4	−58.7 (3)	C26—C27—C28—C23	57.9 (5)
C2—C1—C5—Fe1	58.5 (2)	C24—C23—C28—C27	−58.8 (4)
Pd1—C1—C5—Fe1	−135.3 (4)	P1—C23—C28—C27	169.4 (3)
C3—C4—C5—C1	0.7 (4)	C17—P1—C29—C30	169.9 (2)
Fe1—C4—C5—C1	59.0 (2)	C23—P1—C29—C30	−78.0 (3)
C3—C4—C5—Fe1	−58.3 (3)	Pd1—P1—C29—C30	40.4 (3)
C2—Fe1—C5—C1	−38.9 (2)	C17—P1—C29—C34	−18.8 (4)
C3—Fe1—C5—C1	−83.4 (2)	C23—P1—C29—C34	93.4 (3)
C9—Fe1—C5—C1	−164.6 (5)	Pd1—P1—C29—C34	−148.2 (3)
C7—Fe1—C5—C1	77.6 (3)	C34—C29—C30—C31	−4.5 (5)
C10—Fe1—C5—C1	160.6 (3)	P1—C29—C30—C31	168.2 (3)
C8—Fe1—C5—C1	44.3 (6)	C29—C30—C31—C32	0.4 (6)
C4—Fe1—C5—C1	−120.4 (3)	C30—C31—C32—C33	2.3 (7)
C6—Fe1—C5—C1	118.7 (3)	C31—C32—C33—C34	−0.8 (7)
C2—Fe1—C5—C4	81.4 (3)	C32—C33—C34—C29	−3.4 (6)
C3—Fe1—C5—C4	37.0 (2)	C32—C33—C34—C35	177.1 (4)
C9—Fe1—C5—C4	−44.3 (6)	C30—C29—C34—C33	5.8 (5)
C7—Fe1—C5—C4	−162.0 (3)	P1—C29—C34—C33	−165.1 (3)
C10—Fe1—C5—C4	−79.0 (3)	C30—C29—C34—C35	−174.8 (3)
C8—Fe1—C5—C4	164.6 (5)	P1—C29—C34—C35	14.3 (6)
C6—Fe1—C5—C4	−120.9 (3)	C33—C34—C35—C40	80.8 (5)
C1—Fe1—C5—C4	120.4 (3)	C29—C34—C35—C40	−98.7 (5)
C2—Fe1—C6—C7	−42.9 (6)	C33—C34—C35—C36	−94.5 (5)
C3—Fe1—C6—C7	160.3 (4)	C29—C34—C35—C36	86.0 (5)
C9—Fe1—C6—C7	80.9 (4)	C42—N3—C36—C37	−113.7 (5)
C10—Fe1—C6—C7	119.5 (5)	C43—N3—C36—C37	18.8 (6)
C8—Fe1—C6—C7	37.4 (3)	C42—N3—C36—C35	67.7 (5)
C4—Fe1—C6—C7	−163.2 (3)	C43—N3—C36—C35	−159.8 (4)
C5—Fe1—C6—C7	−120.8 (3)	C40—C35—C36—C37	3.1 (6)
C1—Fe1—C6—C7	−78.3 (3)	C34—C35—C36—C37	178.4 (4)
C2—Fe1—C6—C10	−162.4 (5)	C40—C35—C36—N3	−178.3 (4)
C3—Fe1—C6—C10	40.7 (7)	C34—C35—C36—N3	−3.0 (6)
C9—Fe1—C6—C10	−38.7 (4)	N3—C36—C37—C38	179.6 (4)
C7—Fe1—C6—C10	−119.5 (5)	C35—C36—C37—C38	−1.7 (7)
C8—Fe1—C6—C10	−82.1 (4)	C36—C37—C38—C39	−0.5 (8)
C4—Fe1—C6—C10	77.3 (4)	C37—C38—C39—C40	1.3 (8)
C5—Fe1—C6—C10	119.7 (3)	C38—C39—C40—C35	0.1 (7)
C1—Fe1—C6—C10	162.2 (3)	C36—C35—C40—C39	−2.3 (6)
C10—C6—C7—C8	−1.0 (5)	C34—C35—C40—C39	−177.9 (4)
Fe1—C6—C7—C8	−60.1 (3)		

### Hydrogen-bond geometry (Å, °)

**Table:**

*D*—H···*A*	*D*—H	H···*A*	*D*···*A*	*D*—H···*A*
C3—H3···Cl2^i^	0.98	2.78	3.671 (5)	151
C16—H16C···Cg1^ii^	0.96	2.68	3.544 (6)	151

Symmetry codes: (i) −*x*+1, −*y*+1, −*z*; (ii) −*x*+1, −*y*+2, −*z*.
